# Peritoneal Dialysis is Associated With A Better Survival in Cirrhotic Patients With Chronic Kidney Disease

**DOI:** 10.1097/MD.0000000000002465

**Published:** 2016-01-29

**Authors:** Che-Yi Chou, Shu-Ming Wang, Chih-Chia Liang, Chiz-Tzung Chang, Jiung-Hsiun Liu, I-Kuan Wang, Lien-Cheng Hsiao, Chih-Hsin Muo, Chi-Jung Chung, Chiu-Ching Huang

**Affiliations:** From the Division of Nephrology and Kidney Institute, Department of Internal Medicine (C-CY, W-SM, L-CC, C-CT, L-JH, W-IK, H-CC); College of Medicine (C-CY, W-SM, L-CC, C-CT, L-JH, W-IK, H-LC, H-CC); Division of Cardiology, Department of Internal Medicine China Medical University Hospital (H-LC); Department of Public Health (M-CH); Management Office for Health Data, China Medical University and Hospital, 91 Hsueh-Shih Road (M-CH); Department of Health Risk Management, College of Public Health (C-CJ); and Department of Medical Research, China Medical University Hospital, Taichung 40402, Taiwan (C-CJ).

## Abstract

Peritoneal dialysis (PD) can be an ideal treatment in cirrhotic patients with ascites and chronic kidney disease stage 5 (CKD 5D) who require dialysis. The survival of cirrhotic patients with CKD 5D on PD, however, is not clear. We compared the survival of cirrhotic patients with CKD 5D on PD and the survival of those on HD.

Two datasets including a cohort study of China Medical University Hospital (CMUH) from 2004 to 2013 and the Longitudinal National Health Insurance Database for Catastrophic Illness Patients (LHID-CIP) of Taiwan from 1996 to 2011 were analyzed. The survival of cirrhotic patients on PD and the propensity score matched cirrhotic patients on HD were analyzed using Cox proportional hazards regression.

In CMUH cohort of 85 PD and 340 HD patients, the all-cause mortality was lower in PD patients compared to it in HD patients (hazard ratio [HR]: 0.48, 95% confidence interval [CI]: 0.31–0.74, *P* < 0.01) after adjustments for confounders. The severity of liver cirrhosis defined by Child–Turcotte–Pugh (CTP) class (*P* < 0.01) was independently associated with all-cause mortality. The model for end-stage liver disease (MELD) score, however, was not associated with all-cause mortality.

In the LHID-CIP cohort of 285 PD and 1140 HD patients, the HR of all-cause mortality in PD patients was 0.61 (95% CI: 0.47 – 0.79, *P* < 0.01), as compared with HD patients.

PD in cirrhotic patients who need dialysis is associated with lower all-cause mortality than HD is. This association is independent of patients’ comorbidity, severity of liver cirrhosis, and serum albumin levels.

## INTRODUCTION

The treatment for cirrhotic patients with chronic kidney disease stage 5 who required dialysis (CKD 5D) is complicated by the decreased effective intravascular volume and the subsequential hemodynamic instability.^1,2^ Hemodialysis (HD) associated hypotension is a common complication ^3^ and is associated with higher mortality in patients with CKD 5 who require dialysis.^4^ Multiple techniques are currently available to prevent hypotension during HD including thermal balance,^5^ biofeedback systems,^6^ and midodrine.^7^ However, HD-associated hypotension remains a common complication in cirrhotic patients on HD.

Fluid removal is slower in peritoneal dialysis (PD) and the risk of dialysis-associated hypotension is lower in PD.^8^ There were 2 clinical observation studies in cirrhotic patients with CKD 5D on PD^9,10^ in the literature. None of these studies compared the survival of cirrhotic patients on PD and HD. In this study, we compared the survival of cirrhotic patients with CKD 5D on PD and the survival of those on HD. As the basal characteristics of PD patients were usually different from HD patients, propensity score matching was used in this study.

## METHODS

### Dataset Sources

The 2 analytic datasets in this study were obtained from China Medical University Hospital (CMUH) and the Bureau of National Health Insurance (BNHI). One dataset is a retrospective cohort study from CMUH from 2004 to 2013. All clinical values, severity of liver cirrhosis, comorbidities, and outcomes were from the review of medical records. This analysis of the CMUH dataset was proven by the IRB of CMUH (DMR99-IRB-301) and the IRB waived the need for informed consent for the review of medical records. The other dataset is the Longitudinal Health Insurance Database for Catastrophic Illness Patients (LHID-CIP) of the Taiwan National Health Research Institute, which was released by the BNHI. The LHID-CIP included all medical records from 1996 to 2011. The International Classification of Disease Revision, 9th Clinical Modification (ICD-9-CM) was used for the diagnosis codes. The analysis of the LHID-CIP dataset was proven by the institutional review board (IRB) of CMUH (CMUH 102-REC3–039).

### Study Participants and Outcome Measures From the CMUH Cohort

**FIGURE 1:**
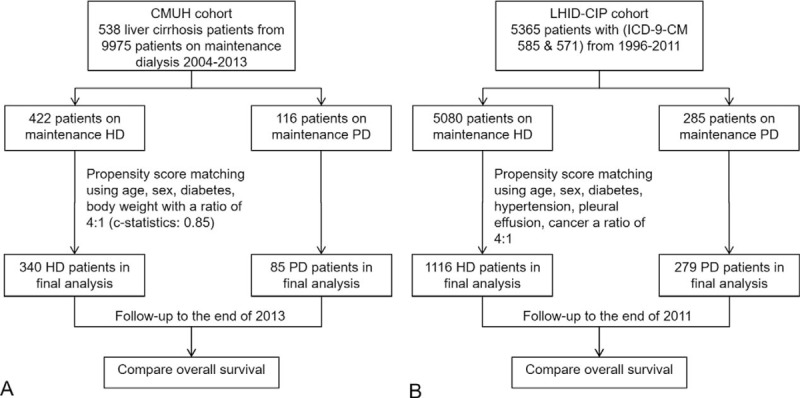
Flowchart of study design. CMUH = China Medical University Hospital, HD = hemodialysis, LHID-CIP = Longitudinal National Health Insurance Database for Catastrophic Illness Patients, PD = peritoneal dialysis.

All CKD 5D patients who received HD or PD for >3 months in CMUH from 2004 to 2013 were included. Of 9975 chronic dialysis patients, 538 cirrhotic patients had liver cirrhosis at the start of dialysis, wherein 116 patients were treated with PD and 422 patients were treated with HD. Forty-seven patients who received hemodiafiltration were not included because none of them had liver cirrhosis. All PD patients received continuous ambulatory peritoneal dialysis and none of these patients had automated peritoneal dialysis. A flowchart of the study design in detail is shown in Figure 1 A. A propensity score-matching scheme at a rate of 1:4 was applied according to age, gender, diabetes, and body weight (c statistics: 0.81). Then, a total of 85 PD patients and 340 HD patients were analyzed in this study. All patients were prospectively followed to the end of 2013 or the date of all-cause death, the date of transfer to kidney transplant, the date of transfer to HD in PD patients, the date of transfer to PD in HD patients, or the date of transfer to another hospital. The primary kidney disease was diagnosed by the primary care physician at the start of dialysis and the diagnosis included diabetes, hypertension, chronic glomerular nephritis, and hepatorenal syndrome. In addition, patient comorbidity, the hemoglobin, serum creatinine, and albumin levels were recorded. The serum albumin, total bilirubin, prothrombin time, international normalized ratio, a history of ascites, a history of hepatic encephalopathy, and causes of liver cirrhosis of each patient were recorded by reviewing the medical records before the start of dialysis. The researchers who collected the data were blind to the analysis and the aims of this study. The Child–Turcotte–Pugh (CTP) classification (CTP class, A: 5–6, B: 7–9, and C: 10–15 points in CTP score) and model for end-stage liver disease (MELD, 1: < 9, 2: 10–19, 3: 20–29, 4: 30–39, 5: ≥ 40 points) score were calculated accordingly.^11,12^ Diabetes mellitus was defined as the use of insulin, a hypoglycemic agent, or a fasting plasma glucose level of 126 mg/dL or more.^13^ Hypertension was defined as the intake of antihypertensives without regard to the actual measurement of blood pressure, or a systolic blood pressure (SBP) reading >140 mm Hg or a diastolic blood pressure (DBP) reading >90 mm Hg.^14^ Cardiovascular disease was defined as 1 or more of the following: myocardial infarction, ischemic stroke, angina pectoris, transient cerebral ischemia, or peripheral vascular disease that did not include occlusion of the arterio–venous fistula.^15^ eGFR was calculated using the Modification of Diet in Renal Disease (MDRD) formula.^16^ The Charlson comorbidity index ^17^ was calculated based on the comorbidity diagnosed by the primary care physician at the start of dialysis. The body mass index (BMI), hemoglobin, creatinine, eGFR, and serum albumin were recorded at the start of dialysis. Kt/V, SBP, and DBP within 3 months after the start of dialysis were recorded. If the patients had >2 available values of these measurements, the average values were used in the analysis.

### Study Participants and Outcome Measures From the LHID-CIP Cohort

For the LHID-CIP cohort study, we identified 5365 patients (5080 HD and 285 PD) that were newly diagnosed cases of ESRD (ICD-9-CM code 585) with liver cirrhosis (ICD-9-CM code 571) from 1996 to 2011. The diagnosis date was defined as the index date. The ESRD group was divided into the HD and PD subgroups. The cause of liver cirrhosis was defined as alcohol-related (ICD-9-CM code 571.2), hepatitis B-related (ICD-9-CM codes V02.61 and 070.2–070.33), and hepatitis C-related (ICD-9-CM codes V02.62 and 070.41, 070.44, 070.54, 070.70, and 070.71). Baseline comorbidities included diabetes (250), hypertension (401–405), pleural effusion (511), and any type of cancer (140–239. These comorbidities were defined to have >3 medical visits. The Charlson comorbidity index ^17^ was calculated based on the ICD-9-CM codes of comorbidity at the start of dialysis. A propensity score matching approach was used because the general condition of the PD patients was usually better than that of the HD patients at the initiation of dialysis.^18^ The variables used in the propensity score were age, gender, and Charlson comorbidity index at a rate of 1:4 for analysis. The c statistics of the propensity score matching was 0.65. All study subjects were followed from the index date of dialysis to the date of all-cause death, the date of transfer to HD in PD patients, the date of transfer to PD in HD patients, or 31 December 2011. A flowchart of the study design in detail is shown in Figure 1 B.

### Statistical Analysis

Data are reported as the mean ± standard deviation, median (interquartile range), or frequency (percentage), as appropriate. All continuous variables were tested for normality using the skewedness and kurtosis test. Data was analyzed using the *t* test for normally distributed variables, the Kolmogorov–Smirnov test for non-normalized variables, or the chi-squared test for categorical variables. The primary end point was all-cause mortality. The censoring events were transfer to PD in HD patients, transfer to HD in PD patients, transfer to kidney transplantation, and transfer to other hospitals. The mortality rate was defined as the number of patients who died divided by the total follow-up person-years (expressed as per 1000 person-years). The incidence rate ratio (IRR) of all-cause mortality was analyzed by Cox proportional hazard regression. The hazard ratio (HR) and 95% confidence intervals (CIs) were calculated. Confounders including age, gender, primary kidney disease, cause of liver cirrhosis, comorbidity index, CTP score, MELD score, Kt/V, hemoglobin, creatinine, eGFR, and albumin were first analyzed using univariate Cox regression model. Variables with *P* < 0.05 were further analyzed using the multivariable Cox regression. All analyses were performed using Stata version 12 SE (StataCorp, TX) or the SAS software, version 9.3 (SAS Institute, Cary, NC). Values with *P* < 0.05 were considered statistically significant.

## RESULTS

### Comparison of Mortality Between PD and HD Patients From CMUH Cohort

The CMUH cohort included 85 PD patients and 340 propensity score matched HD patients. The clinical characteristics at the start of dialysis were similar between PD patients and HD patients (Table 1). The average age was 62.3 ± 16.6 years in PD patients and 62.6 ± 15.3 years in HD patients. Hepatitis B and hepatitis C infection were the major causes liver cirrhosis in both HD and PD patients. Hepatorenal syndrome was the primary kidney disease in 37 (10.8%) HD patients and 6 (7.1%) PD patients. The Charlson comorbidity index was not different between PD patients (4.2 ± 1.1) and HD patients (4.3 ± 1.1, *P* = 0.65). The average CTP score was not significantly different in PD (6.2 ± 4.3) and HD patients (6.1 ± 4.5, *P* = 0.29). The percentages of patients who had CTP class A, B, and C were 56.5%, 20%, and 23.5% in PD patients and 58.5%, 17.4%, and 24.1% in HD patients, respectively. The MELD score was 30.1 ± 2.6 in PD patients and 29.5 ± 3.1 in HD patients (*P* = 0.01). The percentage of patients who had an MELD score 10 to 19, 20 to 29, 30 to 39, and ≥ 40 were 1.8%, 72.9%,25%, 0.3% in HD patients and 0%, 61.2%, 38.8%, and 0% in PD patients. The, systolic blood pressure, diastolic blood pressure, hemoglobin, creatinine, albumin, and eGFR levels at the initiation of dialysis were not different in PD and HD patients.

**TABLE 1:**
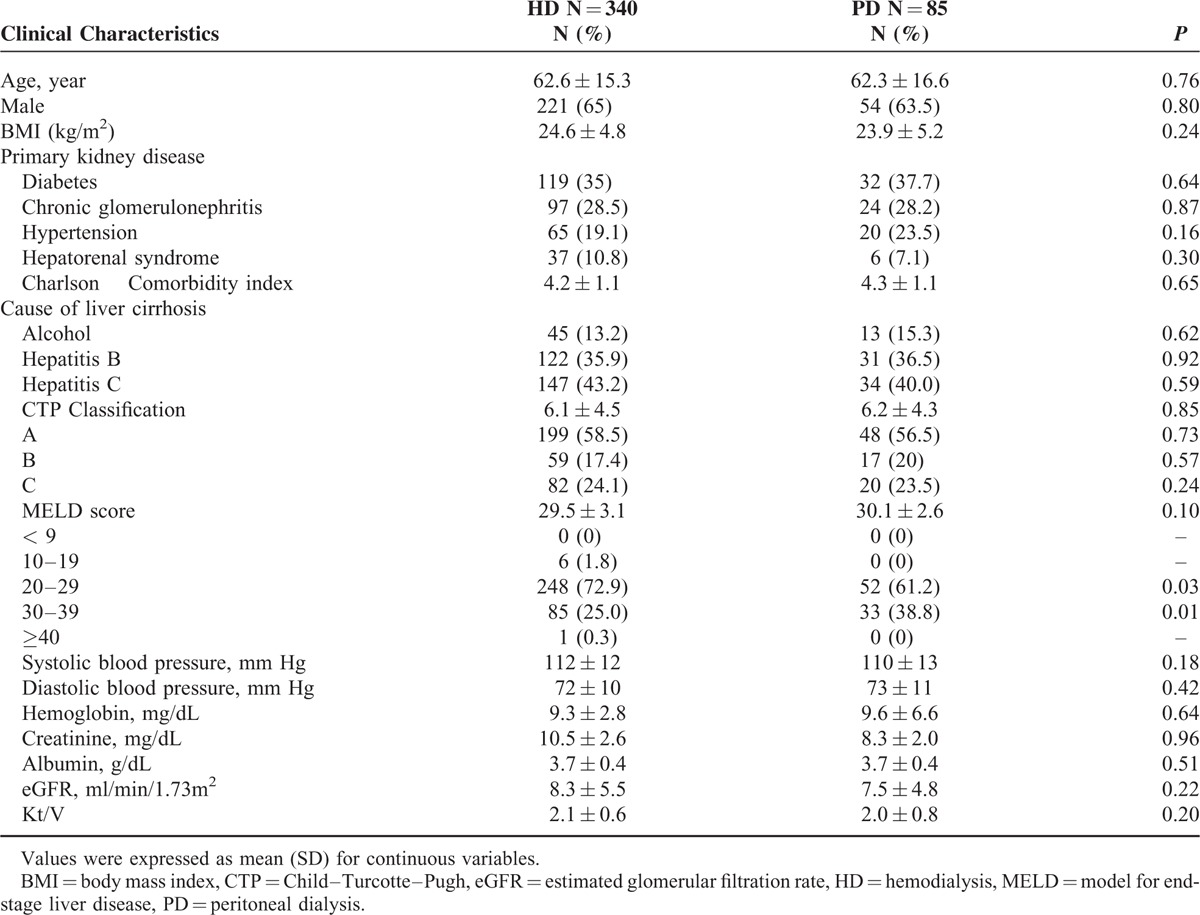
Baseline Characteristics of Liver Cirrhosis Patients With 85 PD and 340 Propensity Score Matched HD From the CMUH Cohort

**FIGURE 2:**
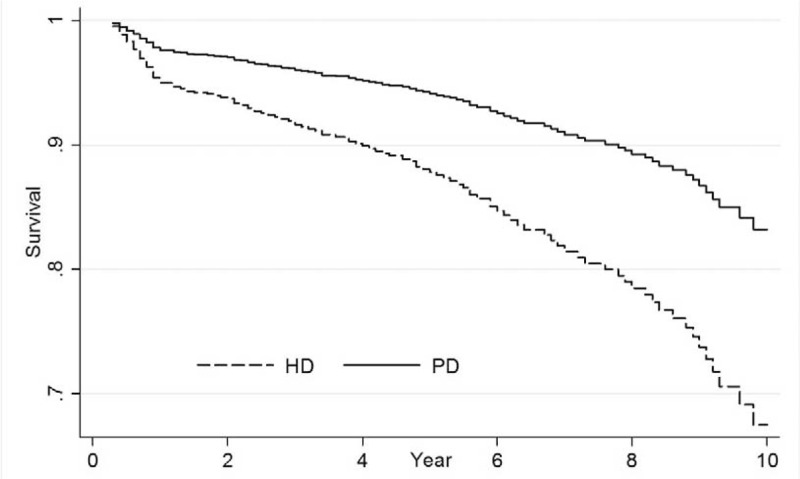
Survival curve of cirrhotic patients on peritoneal dialysis or hemodialysis with adjustments for confounders in China Medical University Hospital Cohort.

After an average of 6-year follow-up, the mortality rate was 39.2 ± 13.2 per 1000 patient-years in PD patients and 69.1 ± 11.9 per 1000 patient-years in HD patients. PD patients had a better survival than HD patients (Figure 2). Of 85 PD patients, 2 patients were transferred to HD because of pleural effusion, 1 patient was transferred to HD because of recurrent peritonitis, and 3 patients received kidney transplantation. Of 340 HD patients, 6 patients were transferred to PD because of malfunction catheter or vascular access infection, 25 patients were transferred to other hospitals, and 9 patients had kidney transplantation. Two models of multivariable Cox regression (Table 2) were generated to compare the survival of cirrhotic patients who received PD or HD because CTP score and MELD score were highly correlated. CTP class was used in model 1 and MELD score was used in model 2. PD was associated with lower all-cause mortality in model 1 (HR: 0.48, 95% CI: 0.31–0.74, *P* < 0.01) and in model 2 (HR: 0.54, 95% CI: 0.34–0.83, *P* = 0.02). CTP class was significantly associated with increased all-cause mortality with an HR of 8.92 (95% CI: .73–11.82, *P* < 0.01) for every 1 class higher of CTP classification. Serum albumin was independently associated with lower all-cause mortality with an HR of 0.62 (95% CI: 0.42–0.92, *P* = 0.01) for every 1 g/dL higher in serum albumin. Patients with hepatorenal syndrome as the primary kidney were associated with increased all-cause mortality in model 1 (HR: 1.82, 95% CI: 1.03–3.21, *P* = 0.01), but not in model 2.

**TABLE 2:**
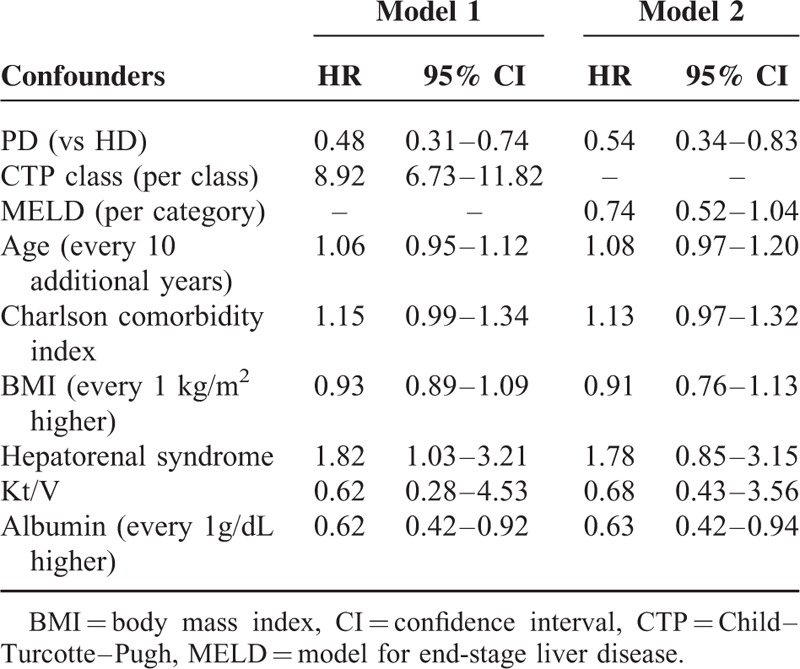
Hazard Ratio of Mortality of Prognostic Factors in Multivariable Cox Proportional Hazard Regression From the CMUH Cohort

### Comparison of Mortality Between PD and HD Patients From LHID-CIP Cohort

**FIGURE 3:**
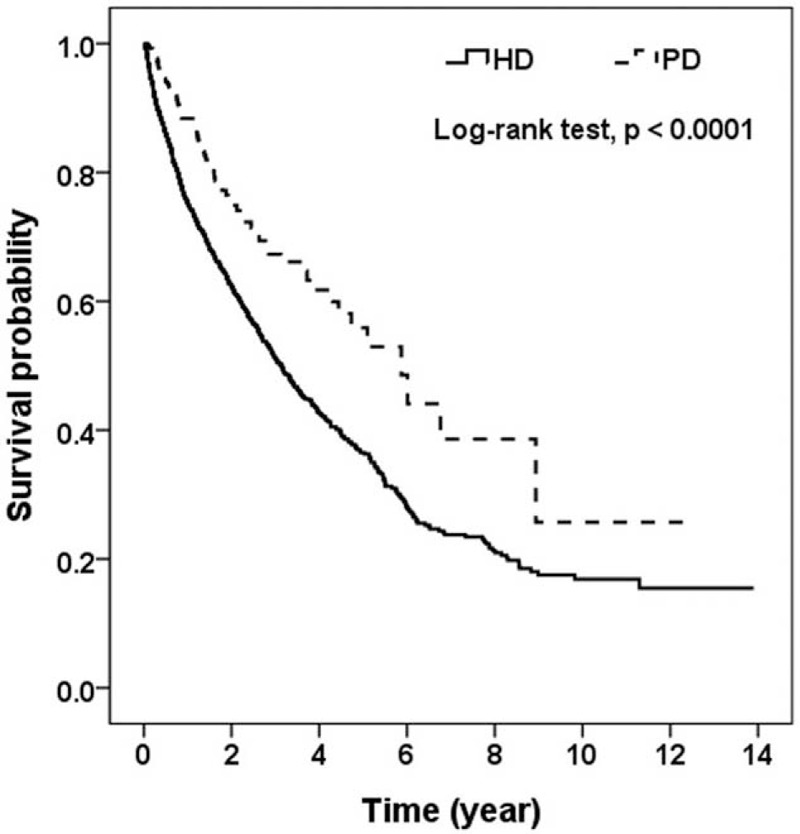
Survival curve of cirrhotic patients on maintenance peritoneal dialysis or hemodialysis in Longitudinal National Health Insurance Database for Catastrophic Illness Patients Cohort.

The LHID-CIP cohort consisted of 279 PD and 1116 matched HD patients. The baseline characteristics including patients’ age, gender, causes of liver cirrhosis, and Charlson comorbidity index of PD patients were not significantly different from that of HD after propensity score matching (Table 3). Of 1116 HD patients, 434 (38.9%) patients were >65. Of 279 PD patients, 103 (36.9%) patients were >65. The Charlson comorbidity index was 4.32 ± 2.27 in HD patients and 4.33 ± 2.34 in PD patients. Hepatitis B and hepatitis C infections were the major cause of liver cirrhosis in these patients. This is similar to what we observed in the CMUH cohort. Of the 1395 patients, 68 and 211 deaths occurred in the PD and HD patients. The survival of PD patients was better than that of HD patients (Figure 3). The all-cause mortality rates were 50.6 per 1000 patient-year in PD patients and 109.6 per 1000 patient-year in HD patients (Table 4). The all-cause mortality was significantly lower in PD patients with a HR of 0.61 (95% CI: 0.47–0.79, *P* < 0.01). After stratification by the median value of the propensity score, PD was significantly associated with lower all-cause mortality in patients with high propensity score with an HR of 0.39 (95% CI: 0.25–0.64, *P* < 0.01).

**TABLE 3:**
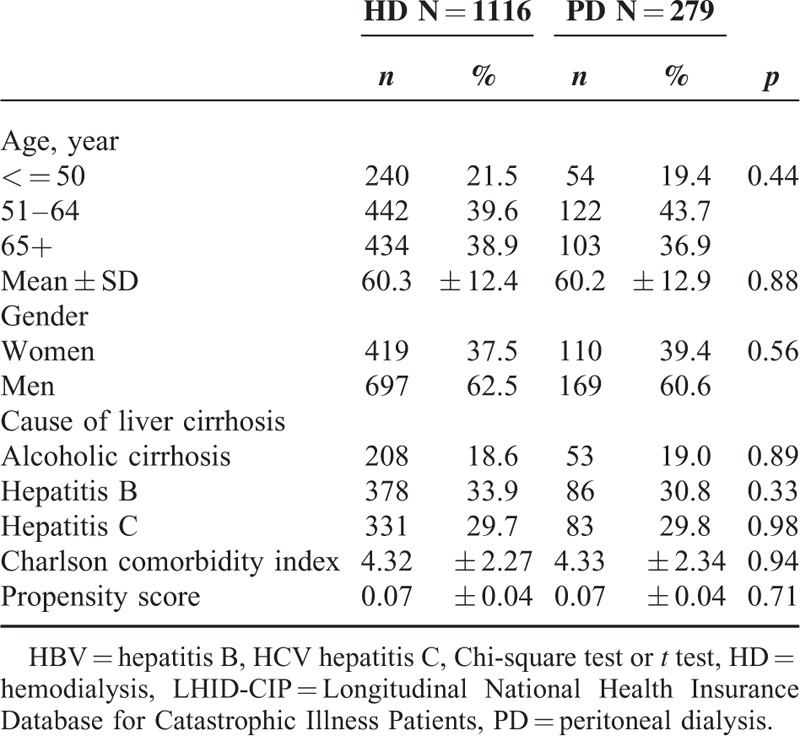
Demographic Characteristics and Comorbidities Between 282 PD and 1128 Propensity Score Matched HD Patients With Cirrhosis From the LHID-CIP Cohort

**TABLE 4:**
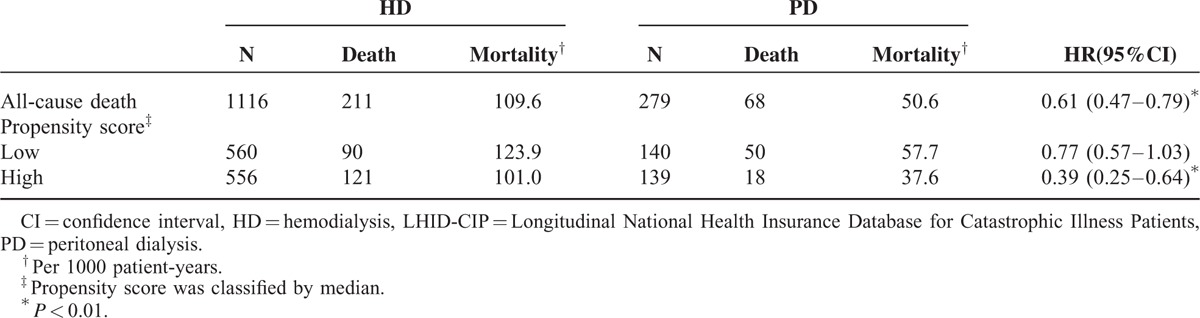
Mortality Risk in PD Patients Compared to HD Patients and Further Stratified by Propensity Score in Cox Proportional Hazard Regression From the LHID-CIP Cohort

## DISCUSSION

This was the first study to compare the survival of PD and HD in cirrhotic patients with CKD 5D that required dialysis. This study clearly showed that PD treatment was associated with lower all-cause mortality than HD treatment. This finding was not only observed in CMUH cohort after the adjustments of clinical confounders including CTP score, comorbidity index, and serum albumin, but also in a nationwide insured population. In addition, the study also demonstrated that the CTP class, but not MELD score, was strongly associated with all-cause mortality in cirrhotic patients who need dialysis. The major strength of this study was the sufficient number of patients for the propensity score matching approach with nationwide data as well as the adjustments for the CTP class with the data from a cohort of a hospital. The lower all-cause mortality in cirrhotic patients on PD may be explained by the lower chance of dialysis-related hypotension and/or the preserve of residual renal function. Hypotension during HD was clearly associated with a higher risk of overall mortality in HD patients.^4^ Among the cirrhotic patients on HD in CMUH, 62% of the patients had episodes of hypotension during HD.^19^ However, only a limited number of patients among the patients on maintenance PD had hypotension. We could not analyze the role of hypotension during dialysis in PD patients because hypotension during dialysis for PD patients has never been defined in the literature. In addition, cardiovascular mortality was the major cause of death (52.4% in HD patients and 48% in PD patients) in cirrhotic patients who received dialysis. Infectious diseases were the cause of death in 7(28%) of 25 PD patients who died and the percentage was not different to that (21.4%) of HD patients (*P* = 0.87, chi-square test). The residual renal function was available in all PD patients but was not available in HD patients. We, therefore, are not able to analyze the impact of residual renal function on all-cause mortality in this study.

The MELD score had been considered as a better tool for the assessment of the survival of cirrhotic patients ^11,20–22^ However, a better correlation for the MELD score as compared with the CTP score was not found in the patient outcomes in the present study. The serum creatinine level was taken into consideration in the MELD score but not in the CTP score. The predicting power of serum creatinine levels on patients’ outcomes may be decreased because of the high serum creatinine levels in our patients or the underestimation of creatinine in cirrhotic patients, whereas the eGFR level may be overestimated.^23–25^ Higher serum creatinine may not be universally associated with poor outcomes in patients on dialysis. A U-shaped correlation between all-cause mortality and patients’ serum creatinine levels was frequently found in patients who need dialysis.^26^ In addition, excessive protein loss from effluent was a major concern in cirrhotic patients on PD. In our clinical practice, most of the cirrhotic patients on PD can tolerate the PD treatment with a minimal decrease of serum albumin. PD treatment also provided a benefit in removal of ascites in these patients and the risk of peritonitis was not increased when compared to the patients without liver cirrhosis.

This study had several limitations. First, the propensity score matching was used to minimize the effect of patients’ basal characteristics on all-cause mortality. The selection of adequate variables to generate an optimal propensity score can be difficult in patients with CKD 5D.^27^ A c statistic of 0.5 to 1.0 was frequently used in the previous studies using propensity score matching. The c statistics of the propensity score matching was 0.65 in LHID-CIP cohort and 0.81 in the CMUH cohort when patients’ body weight was included in the model. Unfortunately, patients’ body weight was not available in the LHID-CIP dataset. Second, the CTP score was unavailable for the LHID-CIP dataset because laboratory data were not recorded in the LHID-CIP data. The percentage of patients with ascites (ICD9-CM code 789.5) was only 0.2%, which suggested underdiagnosed ascites in cirrhotic patients of the LHID-CIP data. To overcome this limitation, we used the data from CMUH cohort and found that the CTP class was independently associated with poor outcomes. Third, the generalizability of our findings based on data from CMUH cohort can be a potential limitation. To overcome this problem, the nationwide data were used to validate the difference of survival in cirrhotic patients on PD as compared with survival in those on HD. Fourth, hypotension during dialysis was proposed as a possible reason for the improved survival in PD patients. However, hypotension during dialysis was well defined in patients on HD but not in patients on PD.^19^ We were unable to investigate the effects of hypotension during dialysis on patient survival in this study. Fifth, the treatment for hypotension during dialysis was not recorded. We were unable to investigate the effects of the treatment for hypotension during dialysis in the patient outcomes.

## SUMMARY

In conclusion, PD may be an ideal treatment for cirrhotic patients with CKD 5D because PD is associated with a lower all-cause mortality in these patients than HD is. The severity of liver cirrhosis defined by CTP class was independently associated with all-cause mortality in cirrhotic patients with CKD 5D. The association was not observed in the MELD score for all-cause mortality in these patients.
